# Cholesterol efflux capacity assay using immobilized liposomes and apolipoprotein B-depleted serum

**DOI:** 10.1042/BSR20190619

**Published:** 2019-06-18

**Authors:** Hima Rani Sapa

**Affiliations:** Department of Medicine, Case Western Reserve University School of Medicine, Cleveland, OH 44106, U.S.A.

**Keywords:** Assay Development, cholesterol, liposomes

## Abstract

Cholesterol efflux capacity (CEC), an important functional step in reverse cholesterol transport, is the main anti-atherosclerotic function of high-density lipoprotein (HDL). Assays that improve the determination of CEC *ex vivo* for clinical applications are constantly explored. In the accompanying article, Horiuchi et al. (*Biosci. Rep.* (2019) **39(4)**, BSR20190213) evaluate the availability of apolipoprotein B-depleted serum for CEC assays. Using their recently developed immobilized liposome-bound gel beads (ILG) method, Horiuchi et al. demonstrate that apolipoprotein B-depleted serum obtained with poly ethylene glycol precipitation enables CEC assays to be easily and accurately introduced into laboratory medicine.

Lack of symptoms for the risk prediction is one of the leading causes of death by cardio vascular disease (CVD). Serum HDL cholesterol (HDL-C) levels have long been shown to be a biomarker of CVD risk and is a powerful inverse predictor of CVD. Regular blood work estimating the HDL levels in addition to lipid metabolic profile is a guideline by American Heart Association and the National Cholesterol Education Program before administering drug therapies for coronary heart disease in United States. However, recent studies suggest that despite elevated HDL-C levels being consistent with atheroprotection, the causal relation between HDL levels and cardiovascular disease is not clear [1]. Two clinical trials involving Niacin [2] or Dalcetrapib [3], drugs aimed at raising HDL-C levels, did not result in a clinical benefit despite elevated HDL levels. Thus, elevated HDL-C levels are not always protective against CVD and mortality. This lacuna in knowledge was overcome with identification of cholesterol efflux capacity (CEC) as a new biomarker that characterizes an important functional step in reverse cholesterol transport. Horiuchi et al. [4] approach is a step in the direction to introduce CEC in laboratory medicine.

Cholesterol efflux capacity is an *in vitro* measure of an individual’s ability to efflux cholesterol [5]. When applied to cholesterol donor cells viz*.*, macrophages in an *ex vivo* system that involves apolipoprotein B-depleted serum from the study participants, the assay acts as a good predictor of cardiovascular risk [6]. CEC is inversely associated with the incidence of cardiovascular events [5,7]. This inverse correlation is seen in many studies but not all. In one report a positive association of enhanced cholesterol efflux with increased incident cardiovascular events was seen [8]. The variations in CEC assays indicate the need to understand more about the various functions of HDL to better adapt in a clinical setting. Since CEC assays serve as a biomarker, clinical laboratories would benefit from innovation in developing an effective and quick method especially considering into account high throughput clinical analysis with a large number of patient samples.

The current assay methods of CEC determination in the laboratory involves culturing of cholesterol donor cells *viz.*, macrophages. The *ex vivo* system in a clinical set-up is not ideal as some clinical laboratories, especially in developing countries might lack cell culture, radioisotope handling or ultracentrifugation equipment and facilities. To address this dependency on infrastructure, authors developed a variation of CEC assay using immobilized liposome-bound gel beads (ILGs, Figure 1). Typically, the cultured THP-1, a monocytic cell line, differentiated to macrophages are routinely used for the CEC assay. As a substitute for cultured cells, ILGs are prepared *in vitro* by making fluorescently labeled cholesterol containing liposomes with Sephacryl S-300 gel beads to induce the formation of large multi-lamellar vesicles [9]. Due to lack of standard method(s) for measuring CEC, there is a lot of variation in protocols. The underlying principle in all the protocols, however, is to estimate the measure of movement of cholesterol from donor cells to extracellular acceptor, such as human serum or plasma [10]. Cholesterol efflux from donor cells is a combination of various transporters, namely, ATP binding cassette transporter subfamily A member 1 (ABCA1), ATP binding cassette transporter subfamily G member 1 (ABCG1) and scavenger receptor B type 1 (SRB1). Therefore, cholesterol efflux from a non-cellular source, such as ILGs would decrease the variations. The use of ILGs has one possible limitation, even though authors follow preparation procedure in line with the established protocols, it is possible that differences among gel preparation lots could cause variations. For a successful quality control, repeated measurement of CEC using different ILG lots and/or different storage periods should be performed to assess the variation.

**Figure 1:**
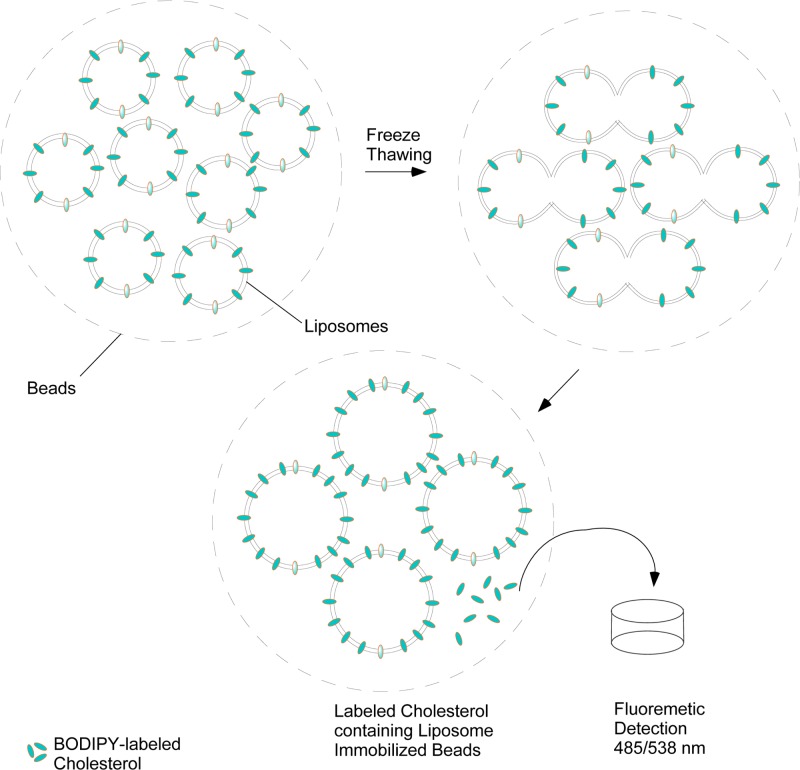
Schematic depiction of liposome entrapment by freeze–thaw cycles The BODIPY labeled cholesterol in the immobilized liposomes act as cholesterol donor.

The next source of variation stems from the choice of cholesterol acceptor in CEC assays. There are three different acceptor media that are used (i) whole serum/plasma; (ii) Apo B depleted serum (BDS); and (iii) pure HDL. Isolation of serum HDL by ultracentrifugation is the most prevalent method. However, the method demands special equipment and is time consuming. In addition, HDL fraction concentrated by ultracentrifugation may not always reflect the concentration of HDL in the serum [11]. The concentrated serum, therefore, needs to be adjusted to the original levels of cholesterol or protein before using for the CEC assay. Hence, usage of much quicker and efficient isolation of HDL fraction that reflects its original concentration in the serum is an important task to correctly measure the cholesterol reverse transport.

To circumvent the ultracentrifugation step for preparation of apolipoprotein B (ApoB)-100 depleted serum, as the cholesterol acceptor, assay developed by Horiuchi et al., use polyethylene glycol (PEG) precipitation. HDL fraction obtained from PEG method (BDS) is indeed an effective alternative instead of using laborious ultracentrifugation method in a clinical setting. Human plasma lipoproteins are routinely fractionated using PEG-6000 [12,13] and widely used at present to prepare BDS. ApoB fraction induces influx than efflux. It is important to note that although apoB-containing lipoproteins are thought to be removed by the PEG precipitation method, a small amount of apoB-containing lipoproteins may remain in BDS as PEG method of precipitation is not affinity based and thus not specific for apoB-containing lipoprotein. However before using PEG method as an alternative method, it is important to understand the quality and quantity of BDS produced by this method comparing to the HDL fraction produced by the ultracentrifugation as the suitability of BDS as a cholesterol acceptor is controversial. Horiuchi et al. [4] evaluated by multiplying CEC obtained using fixed amounts of HDL by cholesterol concentration to HDL-C levels in the serum. Authors observed significant correlation, with Pearson correlation coefficient of 0.633 between the two CECs. Next, to evaluate the compounding effects of proteins present in the serum on the assay results, authors compared BDS-CEC of serum samples reconstituted at different five concentration levels with that of serum proteins with five levels of HDL-C. In such a comparison, authors did not observe any significant change in BDS–CEC in any combination. Based on this authors extend their claim that CEC obtained from BDS reflects not only the function of HDL but also its concentration in serum.

Typically, CEC assay requires cells loaded with excess of radiolabeled cholesterol and a cholesterol acceptor (HDL). Instead of radioisotope labeling the assay developed by Horiuchi et al., employs fluorescently labeled cholesterol using boron-dipyrromethene (BODIPY) dye. Fluorescent intensity is measured by using microplate reader, which is routinely available in a clinical setting, to measure the CEC. Use of fluorescently labeled cholesterol instead of radiolabeled one can be a great choice for working in safe laboratory environment. However, testing the effect of BODIPY-cholesterol concentrations during ILG preparation methodology would be a good option.

One of the limitations of the Horiuchi et al. [4] study is the use of serum from patients whose laboratory data are within the ranges of HDL-C 78 mg/dl with a S.D. of 37. It remains to be seen whether serum with highly abnormal clinical data affects CEC measurement by this new method variation. The Horiuchi et al. study also need to determine a reference interval or cut-off value to estimate the CVD risk of a patient. Using reference sera for CEC assay method could enable to minimize variations between the assays. Addressing these limitations would certainly make the adaptation of CEC method variation one step closer to clinical laboratories.

By changing three steps of CEC assay viz., (i) ILGs for cultured cells; (ii) fluorescently labeled cholesterol; (iii) PEG precipitated HDL, authors demonstrate that it sufficient to estimate CEC with a significant correlation with a conventional assay using foam cells derived from THP-1 macrophages. Here is a holistic approach in which an observation arising from using HDL fraction obtained from the simple PEG method as a cholesterol acceptor in CEC assay. Although there is change in cholesterol safe labeling and the quick method of isolation of HDL fraction, the cultured cells are the only choice for CEC assay until recently. Having artificial cells that is liposome containing fluorescent BODIPY-labeled cholesterol as a substitute for the cultured cells is an effective method in clinical laboratories.

## Abbreviations

ApoB: apolipoprotein B
BDS: Apo B depleted serum
CEC: cholesterol efflux capacity
CVD: cardio vascular disease
HDL: high-density lipoprotein
HDL-c: HDL cholesterol
ILG: immobilized liposome-bound gel bead
PEG: Polyethylene glycol
